# TrDosePred: A deep learning dose prediction algorithm based on transformers for head and neck cancer radiotherapy

**DOI:** 10.1002/acm2.13942

**Published:** 2023-03-03

**Authors:** Chenchen Hu, Haiyun Wang, Wenyi Zhang, Yaoqin Xie, Ling Jiao, Songye Cui

**Affiliations:** ^1^ Institute of Radiation Medicine Chinese Academy of Medical Sciences and Peking Union Medical College Tianjin China; ^2^ Shenzhen Institutes of Advanced Technology Chinese Academy of Sciences Shenzhen China; ^3^ Department of Medical Physics Memorial Sloan Kettering Cancer Center New York USA

**Keywords:** deep learning, radiation therapy, treatment planning

## Abstract

**Background:**

Intensity‐Modulated Radiation Therapy (IMRT) has been the standard of care for many types of tumors. However, treatment planning for IMRT is a time‐consuming and labor‐intensive process.

**Purpose:**

To alleviate this tedious planning process, a novel deep learning based dose prediction algorithm (TrDosePred) was developed for head and neck cancers.

**Methods:**

The proposed TrDosePred, which generated the dose distribution from a contoured CT image, was a U‐shape network constructed with a convolutional patch embedding and several local self‐attention based transformers. Data augmentation and ensemble approach were used for further improvement. It was trained based on the dataset from Open Knowledge‐Based Planning Challenge (OpenKBP). The performance of TrDosePred was evaluated with two mean absolute error (MAE) based scores utilized by OpenKBP challenge (i.e., Dose score and DVH score) and compared to the top three approaches of the challenge. In addition, several state‐of‐the‐art methods were implemented and compared to TrDosePred.

**Results:**

The TrDosePred ensemble achieved the dose score of 2.426 Gy and the DVH score of 1.592 Gy on the test dataset, ranking at 3rd and 9th respectively in the leaderboard on CodaLab as of writing. In terms of DVH metrics, on average, the relative MAE against the clinical plans was 2.25% for targets and 2.17% for organs at risk.

**Conclusions:**

A transformer‐based framework TrDosePred was developed for dose prediction. The results showed a comparable or superior performance as compared to the previous state‐of‐the‐art approaches, demonstrating the potential of transformer to boost the treatment planning procedures.

## 1 INTRODUCTION

Cancer is a leading cause of death worldwide, with an estimated 19.3 million new cases and nearly 10 million deaths in 2020.^1^ Over the past decades, intensity‐modulated radiation therapy (IMRT) has been the standard treatment protocol for various treatment sites, as it allows a conformal dose distribution for target volumes with complex shapes.^2^ By using IMRT, beams from different directions are divided into a set of small beamlets, and the intensity of each beamlet is modulated to achieve prescribed dose for the planning target volumes (PTVs) while sparing the organs at risk (OARs). However, treatment planning for IMRT is a time‐consuming and labor‐intensive process, as planners need to repeatedly adjust a number of parameters in treatment planning system (TPS) to determine the intensities of beamlets. Moreover, the quality of a treatment plan highly depends on the expertise and experience of planners.^3^

To streamline the treatment planning process, one solution is multi‐criteria optimization (MCO) which is dedicated to compute a database of Pareto optimal plans for each patient and then select a plan that meets the clinical metrics.^4^, ^5^, ^6^ Another well‐documented solution is knowledge‐based planning (KBP) which generates patient‐specific treatment plans for new patients based on the previously delivered plans. In the last decade, some KBP works aimed at predicting the desirable dose‐volume histograms (DVHs).^7^, ^8^, ^9^ The main limitation of these methods is the lack of 3D information. To overcome this shortcoming, there is a tendency to focus on the voxel‐level dose prediction. The traditional voxel‐level methods devoted to predict 3D dose distributions based on artificial neural networks (ANNs)^10^, ^11^, ^12^ from handcraft features. Although these features are related to anatomical and plan parameters (e.g., PTV volume, distance from OARs), it fails to preserve the spatial relationship between each voxel. Nowadays, convolutional neural networks (CNNs) including U‐net variants,^13^, ^14^, ^15^, ^16^, ^17^, ^18^ residual networks^19^ and generative adversarial networks^20^ have been widely applied for the voxel‐level dose prediction. Wang et al. gave a detailed summary about these methods.^21^

On the other hand, transformer, which is well‐known for its self‐attention mechanism and the capability of learning long‐range dependencies,^22^ has achieved great success in natural language processing (NLP). Motivated by this, several studies tried to introduce the self‐attention mechanisms in CNNs.^23^, ^24^ Recently, transformers have been applied to the vision tasks. Based on the large‐scale data pre‐training, Vision Transformer (ViT), which took as input the sequences of image patches and the position encoding, obtained the promising performance for the image recognition.^25^ Furthermore, many efforts have been devoted to employ transformers on the object detection^26^ and semantic segmentation.^27^ PvT first constructed a four‐stage pyramid structure and proposed a spatial reduction attention.^28^ Swin Transformer calculated the attention within local windows.^29^ CvT utilized the convolutional projection to capture low‐level features.^30^ An in‐depth survey about these vision transformers and self‐attentions can be found in Ref. [31]. For the dose calculation, a recent study framed the particle transport physics as sequence modeling via the transformer encoder and convolutional decoder, improving the speed 33 times faster than the clinical pencil beam algorithm.^32^

In this study, a 3D transformer‐based algorithm was first proposed to predict the treatment dose distributions for the head and neck cancers and evaluated with CNN‐based methods quantitatively. With a database of only 200 patients, the algorithm can be trained from scratch and complete the 3D dose prediction for a patient in seconds.

## 2 METHODS

### 2.1 Dataset

The proposed algorithm was trained and evaluated on the OpenKBP dataset.^33^ It includes 340 head and neck cancer (HNC) patients treated by 6MV IMRT with nine equispaced coplanar beams. Each patient has at least one PTV, at most seven OARs and a dose distribution generated by a 3D generative adversarial network^20^ and a Computational Environment for Radiotherapy Research (CERR).^34^ Each planning CT volume has a fixed dimension of 128×128×128 and an approximate resolution of 3.5 mm × 3.5 mm × 2 mm. The dataset was divided as the same division of the OpenKBP challenge (i.e., patient 1–200 for training, patient 201–240 for validation and patient 241–340 for test).

### 2.2 Architecture of TrDosePred

**FIGURE 1:**
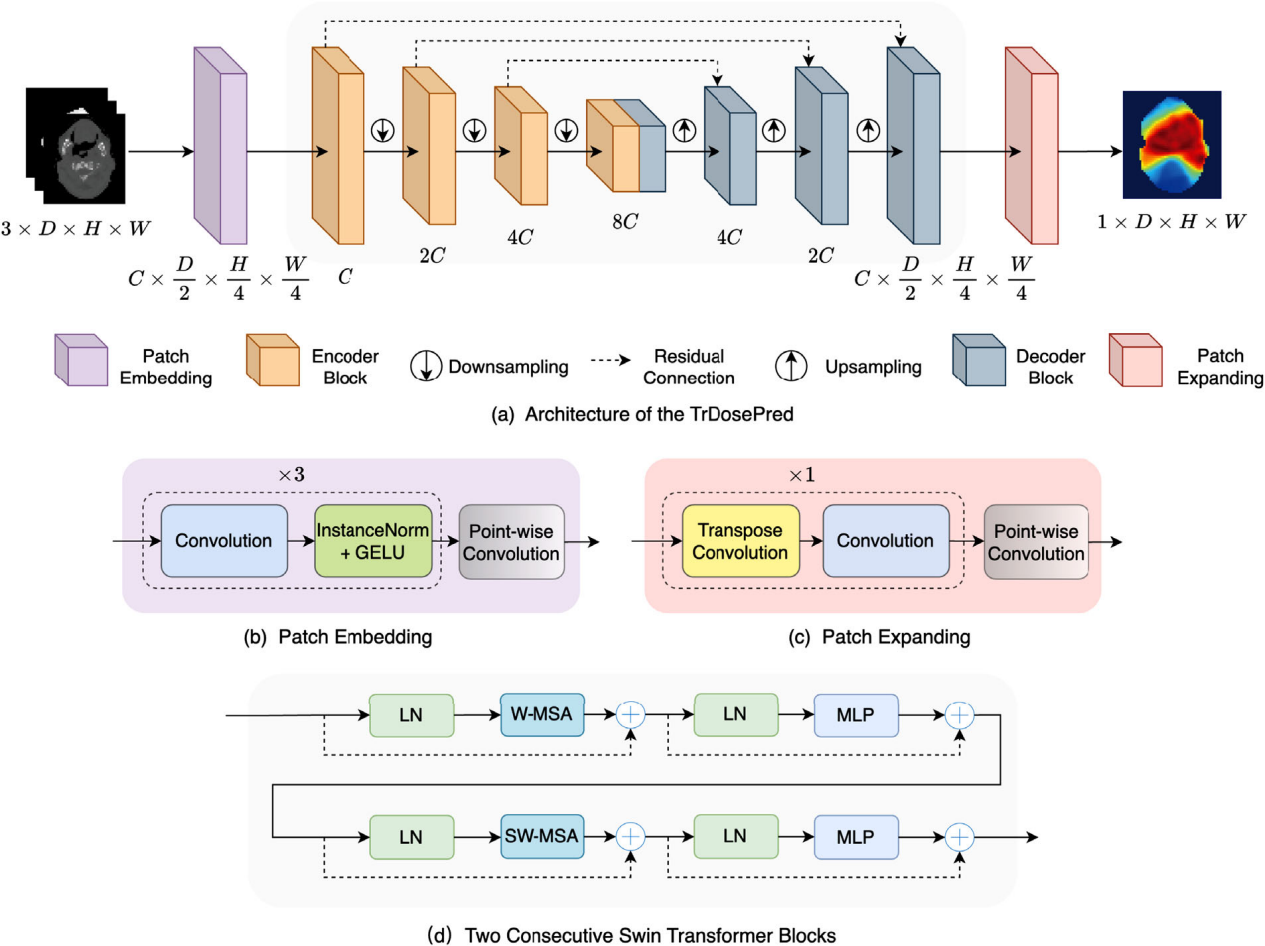
**(a)** The architecture of proposed TrDosePred. With a three‐channel feature of contoured CT as input, patch embedding block first projected it into a sequence of tokens. Transformer‐based encoder and decoder then built the relationship between embedded input features and dose maps. To produce the hierarchical representation, the Downsampling layer halved the feature size D,H,W and doubled the number of channels *C*, while the Upsampling layer operated in reverse. Finally, a patch expanding block was applied to generate the 3D dose distribution. **(b)** Patch Embedding block **(c)** Patch Expanding block **(d)** The illustration of the consecutive swin transformer blocks used in the encoder and decoder. W‐MSA and SW‐MSA are self‐attention modules with regular and shifted window, respectively.

Figure 1a shows the overall architecture of the proposed TrDosePred. With a three‐channel feature of contoured CT as input, a patch embedding block first projected it into a sequence of patch tokens. A transformer‐based encoder and decoder then built the relationship between embedded input features and dose maps. Finally, a simple patch expanding block was applied to generate the 3D dose distribution. These components are elaborated in Section 2.2.1 and Section 2.2.2.

#### 2.2.1 Patch embedding block and expanding block

Traditionally in ViT, the input image was first split and linearly mapped to non‐overlapping patches by patchify convolutions (i.e., stride equals to kernel size) before being fed into the transformer encoder. However, a recent research suggested that the overlapping convolutional stem (i.e., stride smaller than kernel size) used in shallow layers of the vision transformer can remarkably improve the optimization stability and performance.^35^ Motivated by this, a patch embedding block which composed of several stacked overlapping convolution layers, was provided to extract patches from the input volume. As shown in Figure 1b, the patch embedding block consisted of three submodules, each with a 3×3×3 convolution, an Instance Normalization^36^ and a Gaussian Error Linear Units activation function (GELU).^37^ After the third submodule, a point‐wise convolution with 96 filters was used to project the features to embedding tokens. After the patch embedding, the dimension of features was reduced by the factor of 2×4×4.

Symmetrically, a patch expanding block constructed with a 2×4×4 transpose convolution and a 3×3×3 convolution, was applied to recover the resolution of feature maps after the decoder (illustrated in Figure 1c). In the end, a point‐wise convolution was used to generate the dose prediction.

#### 2.2.2 Transformer‐based encoder and decoder

After the patch embedding, the extracted tokens were fed into a U‐shape encoder and decoder, where several 3D swin transformer blocks were heaped. Compared to the vanilla one,^25^ the computation complexity is linear to image size in the swin transformer, which makes it more suitable for the medical image analysis.

Two consecutive 3D swin transformer blocks are elaborated in Figure 1d. Each 3D swin transformer block consists of a window‐based local multi‐head self‐attention (W‐MSA) module and a Multi‐layer Perceptron (MLP) module. To add the locality, a depth‐wise convolution was introduced between the fully connected layers in MLP. In addition, Layer Normalization (LN) and residual connection were adopted before and after each module, respectively. It should be noted that, to establish cross‐window connections, the windows are cyclic‐shifted between two consecutive swin transformer blocks (i.e., SW‐MSA). The computational procedure of two consecutive 3D swin transformer blocks can be formulated as:

(1) $$\begin{array}{cc} & Z_{l}^{\prime }=3DW-MSA\left(LN\left(Z_{l-1}\right)\right)+Z_{l-1}, \end{array}$$

(2) $$\begin{array}{cc} & Z_{l}=MLP\left(LN\left(Z_{l}^{\prime }\right)\right)+Z_{l}^{\prime }, \end{array}$$

(3) $$\begin{array}{cc} & Z_{l+1}^{\prime }=3DSW-MSA\left(LN\left(Z_{l}\right)\right)+Z_{l}, \end{array}$$

(4) $$\begin{array}{cc} & Z_{l+1}=MLP\left(LN\left(Z_{l+1}^{\prime }\right)\right)+Z_{l+1}^{\prime }, \end{array}$$

where $Z_{l}^{\prime }$ and $Z_{l}$ denote the output of the 3D(S)W‐MSA and the MLP module for the $l_{th}$ block.

The attention in each 3D local window can be computed as:

(5) $$\begin{array}{cc} & Attention\left(Q,K,V\right)=SoftMax\left(\frac{QK^{T}}{\sqrt{d_{k}}}+B\right) \end{array}$$

where $Q,K,V\in R^{N_{T}\times d_{k}}$ denote the query, key and value metrics; $N_{T}$ represents the number of tokens in a 3D window; $d_{k}$ is the dimension of the query and key. The values in *B* are token from a bias matrix $\hat{B}\in R^{\left(2D_{w}-1\right)\times \left(2H_{w}-1\right)\times \left(2W_{w}-1\right)}$, where ($D_{w}$, $H_{w}$, $W_{w}$) is the dimension of the 3D local window. Details can be found in Liu et al.^29^

Refer back to Figure 1a, between the encoder and decoder blocks, down‐sampling and up‐sampling layers were inserted respectively. Specifically, 3×3×3 convolutions pre‐activated by a GELU and LN were applied to halve the features and double the number of channels between encoder blocks, while 2×2×2 transpose convolutions were employed to progressively restore the features between decoder blocks. Moreover, residual connections were added between the features extracted by encoder and the counterpart in decoder.

### 2.3 Implementation details

#### 2.3.1 Data pre‐processing

The three‐channel input volume is a concatenation of planning CT, OARs and PTVs. In the planning CT channel, CT values were cropped to range from −1024 to 1500 and then divided by 1000. In the PTV channel, each voxel inside PTVs was assigned to the corresponding prescription dose and normalized by 70 Gy. In the OAR channel, seven OAR masks were labeled by different integers and merged together (1: brain stem, 2: spinal cord, 3: right parotid, 4: left parotid, 5: esophagus, 6: larynx, 7: mandible). To improve the robustness of TrDosePred, data augmentations including random flipping along inferior‐superior and right‐left axes, as well as random translation (at most 20 voxels along each axis) and random rotation (rotation degree is randomly chosen from a list of [$0^{\circ },40^{\circ },80^{\circ },120^{\circ },160^{\circ },200^{\circ },240^{\circ },280^{\circ },320^{\circ }$]) around inferior‐superior axis were applied during the training process.

#### 2.3.2 Training setting

To construct the TrDosePred, the number of swin transformer layers included for each block in encoder and decoder was set to [2, 2, 2, 1] and [2, 2, 2, 2] respectively. A mini‐batch of two samples was applied for each iteration during training. In optimization procedure, mean absolute error was used to calculate the deviation between the clinical and predicted dose distributions. An AdamW optimizer and a cosine annealing scheduler were used (learning rate: $3\times 10^{-4}$, weight decay: $1\times 10^{-4}$). The stopping criterion was 200 epochs.

#### 2.3.3 Ensemble approach

A cross‐validation of five folds was used to obtain an ensemble model. Moreover, for a patient on the test set, test‐time augmentation was applied to get a more robust and accurate prediction.^38^ Specifically, the three‐channel test input was first flipped along (a) right‐left axis, (b) inferior‐superior axis, (c) right‐left and inferior‐superior axes, (d) none, and then fed into each of the model in the ensemble, resulting in a total of 20 intermediate dose predictions. Afterwards, these intermediate predictions were reverted to the original orientations, and averaged to generate the final prediction of the patient.

### 2.4 Comparison with state‐of‐the‐art methods

To evaluate the performance, the results of the top three methods reported in the official OpenKBP challenge paper^33^ were retrieved and compared with the TrDosePred, namely (1) C3D:^16^ a Cascaded 3D U‐net, (2) 3D DCNN:^17^ an ensemble of 3D patch‐based densely connected U‐net with dilated convolutions, and (3) Unet‐ResNet3D:^18^ a U‐net implementation of the pix2pix model with a feature‐based loss.

Furthermore, several cutting‐edge methods were implemented and compared with TrDosePred, including (1) DeepDose:^14^ a 3D variant of U‐net first used for the dose calculation, (2) HD‐Unet:^13^ a hierarchically densely connected 3D U‐net for the dose prediction of HNCs, (3) 2D DCNN:^15^ a 2D densely connected U‐Net with dilated convolutions, (4) Swin‐Unet:^39^ a 2D pure transformer‐based U‐shape architecture primarily used in medical image segmentation. The training setting of these models was carried out under the same conditions of the C3D (batch size: 2, Adam optimizer and cosine annealing scheduler, learning rate: $3\times 10^{-4}$, weight decay: $3\times 10^{-5}$). The same data augmentation and ensemble strategies were used for all models.

In the end, the predictions of the best model will be used to validate and compare the dose distributions and DVH criteria with clinical plans. Two‐sided Wilcoxon test was applied to determine the statistical difference between models. All experiments were conducted on Python 3.9, Pytorch 1.9 and a NVIDIA RTX 3090 GPU.

### 2.5 Ablation study

To investigate the effectiveness of each component, we constructed a baseline based on the modules applied in the Swin Transformer model,^29^ while kept its architecture same as the proposed model. The single TrDosePred was re‐assembled from the baseline step by step and compared the performance quantitatively. The baseline model is denoted as 'SwinTr+PE+PM+TEx', where SwinTr represents the swin transformer blocks used in the encoder and decoder, PE and PM represent the Patchify Embedding and Merging (Downsampling) strategies, and TEx means the Trilinear Expanding (Upsampling) strategy. The single TrDosePred is denoted as 'SwinTr+ConvPE+ConvDown+DeconvEx+DW-MLP'.

## 3 RESULTS

Table 1 summaries the dose score and DVH score of each model over the entire test set. The dose score measures the mean absolute error (MAE) of dose distributions between the prediction and clinical plans.^33^ The DVH score calculates the MAE over five DVH criteria (i.e., *D*_99_, *D*_95_, *D*_1_ for three PTVs and $D_{mean}$, $D_{0.1cc}$ for seven OARs).

**TABLE 1:** Comparison with state‐of‐the‐art methods on the test set through the dose score and the DVH score.

Method		Dose score (Gy)	DVH score (Gy)
OpenKBP Challenge^a^	C3D(1st)	2.429	**1.478**
	3D DCNN(2nd)	2.564	1.704
	Unet‐ResNet3D(3rd)	2.615	1.582
Single Model	DeepDose	2.663	1.741
	HD‐Unet	2.697	1.802
	2D DCNN	2.725	1.620
	Swin‐Unet	2.882	1.757
	TrDosePred(ours)	2.512	1.658
Ensemble	DeepDose	2.558	1.693
	HD‐Unet	2.588	1.680
	TrDosePred(ours)	**2.426**	1.592

^a^ The results of the top methods were retrieved from the official OpenKBP challenge paper^33^

On average, for a patient on the test set, TrDosePred ensemble predicted the dose distribution within 2.32 s (including pre‐processing). It achieved the dose score of 2.426 Gy (3.5% of prescription dose of PTV70), outperforming all implemented methods and the top three solutions of the OpenKBP Challenge. For DVH score, TrDosePred ensemble obtained 1.592 Gy (2.2% of prescription dose of PTV70), ranking 3rd in the listed approaches of Table 1.

Table 2 shows the MAE of all regions of interest (ROIs) for the ensemble models on the test set. The TrDosePred ensemble significantly outperforms the DeepDose and HD‐Unet on the PTV70 and most of the OARs. The DeepDose ensemble achieved smaller MAEs on the PTV63, PTV56, and the HD‐Unet ensemble achieved a smaller MAE on the Larynx. No significant difference was found on these structures than the proposed model.

**TABLE 2:** Comparison with ensemble methods for all ROIs on the test set through the MAE (mean ± standard deviation).

ROIs	DeepDose	HD‐Unet	TrDosePred(ours)
PTV70 (Gy)	1.367^a^ + 0.791	1.475^a^ + 0.806	1.259 + 0.703
PTV63 (Gy)	1.944 + 1.032	1.984 + 1.004	1.979 + 1.111
PTV56 (Gy)	1.663 + 0.820	1.738^a^ + 0.806	1.666 + 0.878
Brain Stem (Gy)	1.500^a^ + 2.126	1.429 + 2.015	1.423 + 2.019
Spinal Cord (Gy)	1.660^a^ + 0.705	1.660^a^ + 0.698	1.556 + 0.608
Right Parotid (Gy)	2.990^a^ + 1.040	2.953^a^ + 1.035	2.766 + 0.942
Left Parotid (Gy)	2.971^a^ + 1.006	2.911^a^ + 0.951	2.749 + 0.996
Esophagus (Gy)	2.620 + 1.436	2.482 + 1.317	2.454 + 1.255
Larynx (Gy)	3.006 + 1.444	2.913 + 1.439	2.918 + 1.556
Mandible (Gy)	3.472^a^ + 1.248	3.475^a^ + 1.241	3.289 + 1.358

^a^ Indicates that values are significantly different than the proposed model (p<0.05).

Table 3 shows the MAE between the clinical plans and the predictions of ensemble models on the test dataset. The TrDosePred ensemble predicted *D*_99_, *D*_95_, *D*_1_ within 1.838 ± 2.383Gy, 1.407 ± 1.964 Gy and 1.474 ± 1.269 Gy while predicted $D_{mean}$, $D_{0.1cc}$ within 1.312 ± 1.442Gy and 1.898 ± 2.185Gy. Significant differences were found for metrics except $D_{0.1cc}$.

**TABLE 3:** Comparison of DVH metrics for ensemble models on the test set through the MAE (mean ± standard deviation).

Models	*D*_99_(Gy)	*D*_95_(Gy)	*D*_1_(Gy)	$D_{mean}$(Gy)	$D_{0.1cc}$(Gy)
DeepDose	2.001^a^ + 2.465	1.494^a^ + 2.003	1.777^a^ + 1.419	1.410^a^ + 1.527	1.894 + 2.162
HD‐Unet	2.023^a^ + 2.436	1.579^a^ + 2.028	1.774^a^ + 1.342	1.323 + 1.465	1.894 + 2.157
TrDosePred	1.838 + 2.383	1.407 + 1.964	1.474 + 1.269	1.312 + 1.442	1.898 + 2.185

^a^ Indicates that values are significantly different than the proposed model (p<0.05).

**FIGURE 2:**
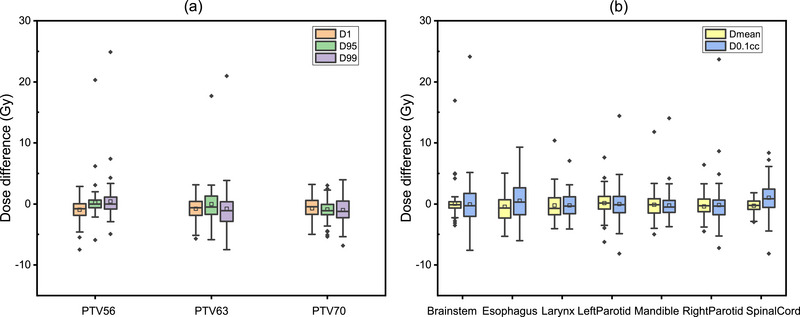
The difference between clinical and TrDosePred ensemble predicted DVH metrics is plotted. **(a)** *D*_1_, *D*_95_, *D*_99_ for PTV56, PTV63 and PTV70, **(b)** $D_{mean}$, $D_{0.1cc}$ for seven OARs including Brainstem, Esophagus, Larynx, Left Parotid, Mandible, Right Parotid and Spinal Cord. The boxes indicate median, mean and interquartile range (IQR). Whiskers extend to 1.5 times the IQR. Outliers are represented by diamonds.

The differences between the clinical and predicted DVH metrics of TrDosePred ensemble are presented in Figure 2. On general, the medians of all metrics were distributed between −1.208 and 0.854 Gy, and the means of all metrics were distributed between −1.022 and 1.010 Gy.

**FIGURE 3:**
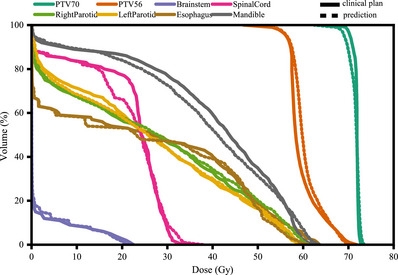
Comparison of the clinical and TrDosePred ensemble predicted DVH curves for a patient in the test dataset. The solid lines represent the clinical plan while dash lines represent the prediction.

**FIGURE 4:**
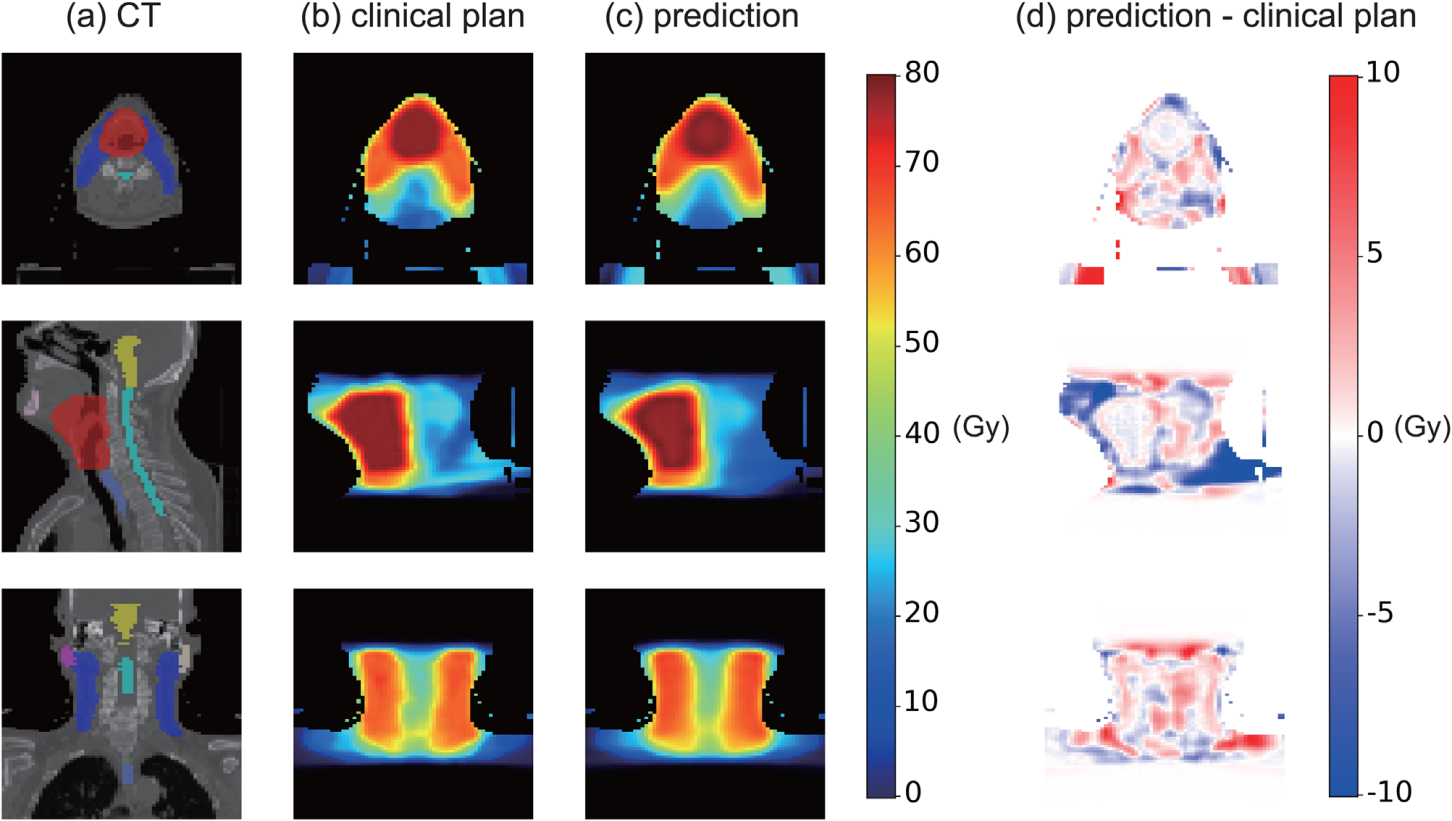
Comparison of the clinical and TrDosePred ensemble predicted dose maps for a patient in the test dataset. **(a)** CT, **(b)** clinical dose, **(c)** the prediction of the TrDosePred ensemble, **(d)** the difference between the prediction and clinical dose (prediction ‐ clinical).

Figure 3 presents the clinical and TrDosePred ensemble predicted DVH curves of a patient randomly selected from the test set. The MAEs of the *D*_99_, *D*_95_, *D*_1_ were 2.006, 1.283, 0.531 Gy on the PTV70 and 0.447, 0.133 Gy, 0.758 Gy on the PTV56. The MAEs of the $D_{mean}$, $D_{0.1cc}$ were 0.680, 1.379 Gy on the spinal cord and 0.102, 1.648Gy on the brain stem. The corresponding dose distributions are shown in Figure 4. Visually, the predicted dose distribution is consistent with the clinical one, as shown in Figure 4b and Figure 4c. Figure 4d shows the differences between the predicted and clinical dose distributions. The mean difference was −0.293Gy on the PTV70 and 0.869Gy on the PTV56.

Table 4 gives out the results of the ablation study. As the process of re‐assembling, both the dose score and DVH score were improved clearly. Especially, a significant improvement was observed when the convolutional sampling strategy was used. ConvPE further boosted the performance by 0.105 and 0.060 Gy for the dose score and the DVH score respectively. DW‐MLP improved the dose score by 0.041 Gy. However, it resulted in a slight decrease on the DVH score and a significant increase on the training time (34 h needed).

**TABLE 4:** Comparison with ablation models. SwinTr is the swin transformer block used to construct the backbone. PE, PM and TEx represent the Patchify Embedding, Patchify Merging and Trilinear Expanding layers used in Ref. [29]. ConvPE refers to our stacked Convolutional Patch Embedding. ConvDown and DeconvEx denote the convolutional Downsampling and Expanding (Upsampling) layers. DW‐MLP means a Depth‐Wise convolution is complemented in the MLP.

Models	Dose score (Gy)	DVH score (Gy)
SwinTr + PE^29^ + PM^29^ + TEx^29^	2.855	2.043
SwinTr + PE + PM + DeconvEx	2.732	1.815
SwinTr + PE + ConvDown + DeconvEx	2.658	1.693
SwinTr + ConvPE + ConvDown + DeconvEx	2.553	1.633
SwinTr + ConvPE + ConvDown + DeconvEx + DW‐MLP	2.512	1.658

## 4 DISCUSSION

In this study, a novel transformer‐based framework TrDosePred, was proposed and evaluated for 3D dose prediction task, based on a group of HNC patients treated by IMRT. Experimental results indicated encouraging performance of TrDosePred for this regression task.

In our experiment, the ensemble of five TrDosePreds on cross‐validation was used and improved the dose score of 3.4% (from 2.512 Gy in the single to 2.426 Gy in the ensemble) against the best single model. To improve the performance, ensemble of models was also applied in the top OpenKBP algorithms. For example, in 3D DCNN, the similar ensemble strategy as in our case was used.^17^ In C3D, a more complex ensemble algorithm was employed. To be specific, five single models were trained and their predictions on the training set were averaged as teacher doses for training a new C3D.^16^ Moreover, the cascaded strategy was explored during the development stage. However, no improvement was observed when two TrDosePred models were cascaded. We assume that the difference was caused by the relative scale of the latter model. Further experiments were not carried out because of the excessive GPU memory cost.

Many of the recent studies on KBP are based on CNNs, which have been limited by the receptive field. To increase the receptive field without loss of resolution, some studies used the dilated convolutions as the basic units.^15^, ^17^ Our approach employed the transformer, whose receptive field can cover the whole input features. This allowed the proposed model to build the long‐range and global connections compared to CNNs. Furthermore, the transformers can make the model robust to perturbations due to their flexible and dynamic receptive fields.^40^

The proposed algorithm can predict 3D dose distributions precisely on the OpenKBP dataset. However, as mentioned in the OpenKBP,^33^ the synthetic dose distributions were used to augment the real clinical ones. Our further investigation indicated that few of the OpenKBP data met the dose constraints of the Radiation Therapy Oncology Group (RTOG) 1016 protocol.^41^ Thus, extended evaluations are required to guarantee the proposed algorithm works well on the real clinical cases. Additionally, in order to generate deliverable plans, the optimization procedure will be required. A popular pipeline for KBP is prediction‐mimicking, which attempts to generate treatment plans as similar as possible to the predicted dose distributions based on voxel‐level, DVH‐level or structure‐level objectives.^19^, ^42^, ^43^ In the future, we could shift our research from the 3D dose prediction to derive optimization parameters of treatment plans.

Some limitations are worth noting. First, it is possible that the capacity of transformer has yet to be fully exploited due to the limited data. Recent studies indicated that combining a few of convolutions with transformers can enjoy both good generalization and capacity, achieving comparable performance against CNNs in the scenario of small dataset and even superior performance as the scale of dataset grows.^30^, ^44^ A further study indicated transformers can outperform CNNs as well based on thousands of medical images.^45^ Therefore, we expect the performance of TrDosePred could be further improved if a larger dataset is given (e.g., thousands of patient data).

Second, a single global dose distribution based loss (i.e., MAE) may not sufficient for optimal DVH metrics. Nguyen et al. suggested that a differentiable DVH loss can improve the domain relevant metrics.^46^ Zimmermann et al. employed a feature‐based loss network, resulting in good performance in DVH score.^18^ Further improvement may be available with incorporating these advanced losses.

## 5 CONCLUSION

In this study, a transformer‐based network, TrDosePred was proposed for dose prediction task. Compared with several existing CNN‐based approaches, TrDosePred can achieve a comparable or superior performance, demonstrating the potential of transformer to accurately and rapidly predict the 3D dose distribution for head and neck cancer patients treated by IMRT.

## AUTHOR CONTRIBUTIONS

Chenchen Hu and Haiyun Wang performed the experiments, the statistical analysis, and drafted the manuscript. Wenyi Zhang and Yaoqin Xie contributed to design of the study and review of analysis. Ling jiao and Songye Cui planned the study and reviewed the manuscript.

## CONFLICT OF INTEREST STATEMENT

The authors have no relevant conflicts of interest to disclose.
